# Mixed-function oxidases and esterases associated with permethrin, deltamethrin and bendiocarb resistance in *Anopheles gambiae s.l.* in the south-north transect Benin, West Africa

**DOI:** 10.1186/1756-3305-6-223

**Published:** 2013-08-06

**Authors:** Nazaire Aïzoun, Rock Aïkpon, Gil Germain Padonou, Olivier Oussou, Frédéric Oké-Agbo, Virgile Gnanguenon, Razaki Ossè, Martin Akogbéto

**Affiliations:** 1 Centre de Recherche Entomologique de Cotonou (CREC), 06 BP 2604, Cotonou, Bénin; 2 Faculté des Sciences et Techniques, Université d’Abomey, Calavi, Bénin

**Keywords:** Susceptibility, Insecticide, CDC bioassay, Synergist, *Anopheles gambiae*

## Abstract

**Background:**

Insecticide resistance monitoring is essential to help national programmers to implement more effective and sustainable malaria control strategies in endemic countries. The current study aimed at an exploring the involvement of detoxifying enzymes in the insecticide phenotype resistance in *Anopheles gambiae s.l.*from Benin, in order to guide future malaria vector control interventions.

**Methods:**

Larvae and pupae of *Anopheles gambiae s.l.* mosquitoes were collected from the breeding sites in Oueme, Atacora and Alibori provinces. CDC susceptibility tests were conducted on unfed female mosquitoes aged 2–5 days old. CDC bioassays were performed with stock solutions of permethrin (21.5 μg per bottle), deltamethrin (12.5 μg per bottle) and bendiocarb (12.5 μg per bottle). CDC biochemical assays using synergists were also conducted to assess the metabolic resistance.

**Results:**

The susceptibility of *Anopheles gambiae* Agbalilame and Kandi populations to permethrin and deltamethrin respectively, increased significantly when synergized by PBO, suggesting an implication of mono-oxygenases in resistance of *Anopheles gambiae s.l.* to pyrethroid. Esterases may play a role in bendiocarb resistance in *Anopheles gambiae* Tanguieta.

**Conclusion:**

Synergists partially restored susceptibility to pyrethroid and carbamate insecticides and might help mitigate the impact of vector resistance in *Anopheles gambiae* Agbalilame, Kandi and Tanguieta populations. However, additional vector control tools are needed to further impact on malaria transmission in such settings.This will improve the implementation and management of future control programs against this important malaria vector in Benin and in Africa in general.

## Background

Malaria is a severe public health problem, causing an estimated 225 million disease cases and 781,000 deaths per year [1]. Most victims are children under five years old living in sub-Saharan Africa [1]. Malaria is transmitted by *Anopheles* mosquitoes, and because there is currently no vaccine available, vector control is one of the most important means of malaria prevention. In most cases, this vector control is carried out through the use of insecticide treated materials or indoor residual spraying. In West Africa, the resistance of *Anopheles gambiae s.l.* to the four major classes of insecticides available for public health has been reported [2-4]. Pyrethroids are the only option for net treatment due to their relative safety for humans at low dosage, excito-repellent properties, rapid rate of knock-down and killing effects [5]. Resistance to this insecticide class is now widespread in the main malaria vectors *Anopheles gambiae s.l* from Benin [6]. Although public health use accounts for only a very small fraction of overall insecticide quantities applied, many vector species of public health importance have already developed resistance to one or more insecticides [7]. Although there are no short term solutions to vector resistance problems, it is important for programme managers to better understand resistance issues and to promote good practices in insecticide based vector control. It is essential to use public health insecticides in such a way that they are safe, effective, and affordable, while taking into account resistance management issues. Vector control programmes need to meet this condition in order to be effective and sustainable.

The main mechanisms that enable insects to resist the action of insecticides can be grouped into two distinct categories: Metabolic resistance is the most common resistance mechanism that occurs in insects. This mechanism is based on the enzyme systems which all insects possess to help them detoxify naturally occurring foreign materials. Three categories of enzymes typically fulfil this function, namely esterases, monooxygenases and glutathione S-transferases [8]. These enzyme systems are often enhanced in resistant insect strains enabling them to metabolise or degrade insecticides before they are able to exert a toxic effect. One of the most common metabolic resistance mechanisms is that of elevated levels, or activity, of esterase enzymes, which hydrolyse ester bonds or sequester insecticides. Nearly all of the strains of *Culex quinquefasciatus* that resist a broad range of organophosphate (OP) insecticides have been found to possess multiple copies of a gene for esterases, enabling them to overproduce this type of enzyme. In contrast, strains of malathion resistant *Anopheles* have been found with non elevated levels of an altered form of esterase that specifically metabolises the OP malathion at a much faster rate than that in susceptible individuals. Metabolic resistance can therefore range from compound specific to very general resistance, affecting a broad range of compounds. Similarly, the level of resistance conferred can vary from low to very high and may differ from compound to compound. Metabolic resistance mechanisms have been identified in vector populations for all major classes of insecticides currently used for vector control, including organophosphates, carbamates, pyrethroids and DDT [7]. While the esterases are associated mainly with resistance to organophosphates and carbamates, high levels of these enzymes have also been involved with resistance to permethrin in *Anopheles gambiae*[9], *Ae. aegypti*[10-12] and *Culex quinquefasciatus*[13]. Synergists can be used to evaluate potential biochemical resistance mechanisms [14]. According to Bingham *et al.*[15], synergists may have an important role to play in the future design of vector control products in an era when alternatives to pyrethroids are scarce.

The second most common resistance mechanism encountered in insects is target site resistance. Insecticides generally act at a specific site within the insect, typically within the nervous system (e.g. OP, carbamate, and pyrethroid insecticides). The site of action can be modified in resistant strains of insects such that the insecticide no longer binds effectively. This results in the insects being unaffected, or less affected, by the insecticide than susceptible insects. For example, the target site for OP and carbamate insecticides is acetylcholinesterase (AChE) in the nerve cell synapses. Several mutated forms of AChE (also called MACE, modified acetylcholinesterase) have been found which result in reduced sensitivity to inhibition by these insecticides; resistance to OPs in Culex spp. e.g. typically results from this mechanism. Similarly, a mutation (known as *kdr*) in the amino acid sequence in the voltage gated sodium channels of nerve cell membranes leads to a reduction in the sensitivity of the channels to the binding of DDT and pyrethroid insecticides. Reduced susceptibility to pyrethroids conferred by *kdr* mutations has been confirmed in *Anopheles gambiae* in West, Central and East Africa [7]. The Beninese National Malaria Control Programme has implemented large-scale and free distribution of LLIN (OlysetNet) since July 2011 throughout the country to increase coverage of LLINs and in addition indoor residual spraying (IRS) with bendiocarb was in progress in the north of the country, thanks to PMI (President’s Malaria Initiative). It is crucial that information on susceptibility to the main insecticides used in public health in Benin and the underlying mechanisms are investigated. This will properly inform control programs of the most suitable insecticides to use and facilitate the design of appropriate resistance management strategies.

The main goal of this study was to determine the susceptibility status, insecticide resistance levels in *Anopheles gambiae* Agbalilame, Tanguieta and Kandi populations to permethrin, bendiocarb, and deltamethrin respectively, and explore the involvement of the detoxifying enzymes in the insecticide phenotype resistance by using synergists.

## Methods

### Study area

**Figure 1:**
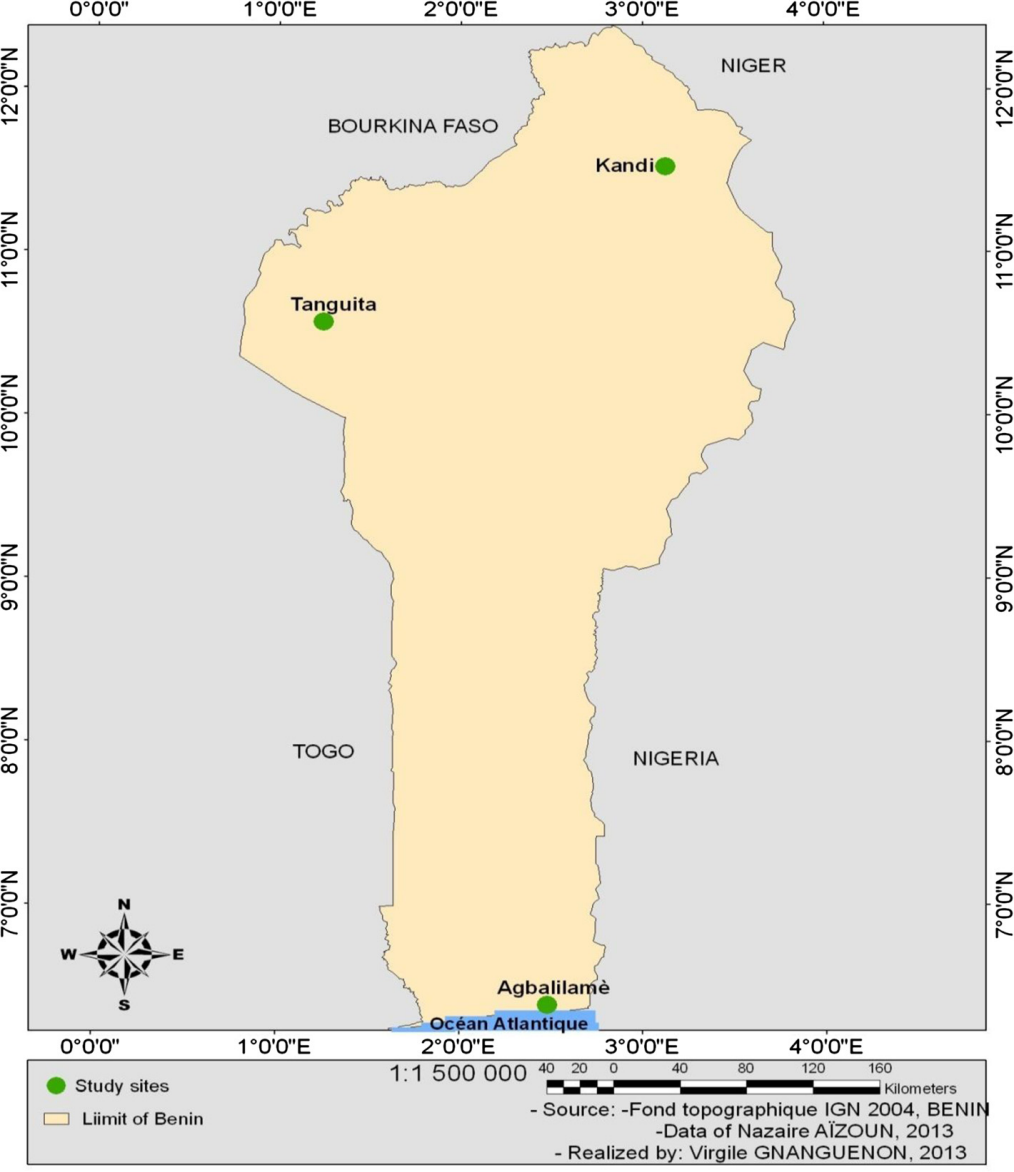
Map of the study area.

The study was carried out in the South of Benin, more precisely in Agbalilame, in the Seme district of Oueme province and in the North of Benin, in the Tanguieta district of Atacora province and in the Kandi district of Alibori province (Figure 1). The choice of the study sites took into account the economic activities of populations, their usual protection practices against mosquito bites, the indoor residual spraying (IRS) in progress in certain of these localities and peasant practices to control farming pests. These factors have an impact on the resistance development in the local vector mosquitoes. Oueme has a climate with four seasons, two rainy seasons (March-July and September-November) and two dry seasons (December-March and August-September).The temperature ranged from 25 to 30°C with the annual mean rainfall, which is between 900 and 1500 mm. Atacora and Alibori have a climate with two seasons, one rainy season (May-October) and one dry season (November- April).The temperature ranged from 23 to 33°C with the annual mean rainfall, which is 1300 mm.

### Mosquito collection

*Anopheles gambiae s.l. *mosquitoes were collected during the rainy seasons (March-July and September-November 2012) across Agbalilame in the Seme district selected in south Benin. *Anopheles gambiae s.l. *mosquitoes were also collected during the rainy season (May-October 2012) across Tanguieta and Kandi districts selected in North Benin. Larvae and pupae were collected on breeding sites using the dipping method. They were then kept in separated labeled bottles related to each locality. The samples were reared up to adult emergence at the CREC(Centre de Recherche Entomologique de Cotonou, Benin) insectary. *Anopheles gambiae* Kisumu, a reference susceptible strain was used as a control for the bioassay tests.Susceptibility tests were carried out following CDC protocols on unfed female mosquitoes aged 2–5 days old reared from larval and pupal collections. All susceptibility tests were conducted in the CREC laboratory at 25+/−2°C and 70 to 80% relative humidity.

### CDC protocol

The principle of the CDC bottle bioassay is to determine the time it takes an insecticide to penetrate an arthropod, traverse its intervening tissues, get to the target site, and act on that site relative to a susceptible control. Anything that prevents or delays the compound from achieving its objective of killing the arthropods contributes to resistance. Diagnostic doses that were applied in the present study were the doses recommended by CDC [16]. These doses were checked on the *Anopheles gambiae* Kisumu susceptible reference strain before being applied to field populations. For *Anopheles gambiae s.l.*, the diagnostic dose of 12.5 μg per bottle for both deltamethrin and bendiocarb was used for a diagnostic exposure time of 30 minutes, whereas the diagnostic dose of 21.5 μg per bottle for permethrin was used for the same diagnostic exposure time. The choice of bendiocarb was justified by its use for Indoor Residual Spraying (IRS) in Atacora, whereas permethrin is the insecticide used on OlysetNets that were distributed free by the NMCP in July 2011 across the entire country. We used deltamethrin, an insecticide of same class as permethrin to check if there was already resistance to this product in Kandi district.

The solutions were prepared and the bottles coated according to the CDC protocol [16]. Fifteen to 20 unfed female mosquitoes aged 2–5 days old were introduced into four

250 ml Wheaton bottles coated with insecticide and one control bottle coated with acetone only. The number of dead or alive mosquitoes was monitored at different time intervals (15,30, 35, 40, 45, 60, 75, 90, 105, 120 minutes). This allowed us to determine the total percent mortality (Y axis) against time (X axis) for all replicates using a linear scale.

### Biochemical assays using synergists

**Figure 2:**
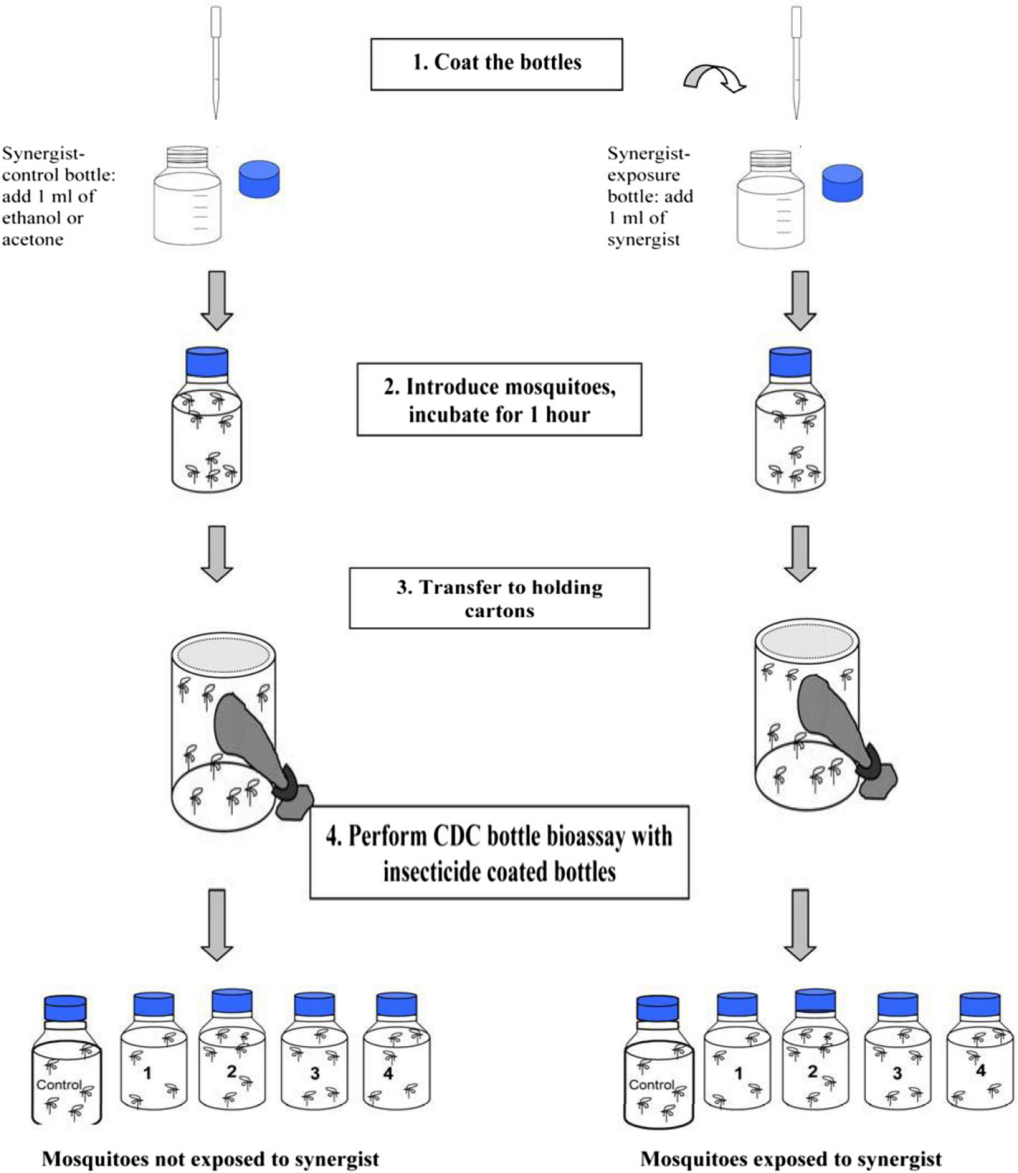
**Diagram for performing the CDC bottle bioassay with synergists **[16]**.**

Synergists were used according to the protocol described by CDC [14,16] following the procedure outlined in Figure 2. Samples that showed high resistance to permethrin and deltamethrin in Agbalilame from the Seme district and in Kandi district respectively were exposed to the effects of the synergist: piperonyl butoxide (PBO) (400 μg/bottle), which inhibits oxidase activity. Samples that showed high resistance to bendiocarb in Tanguieta district were exposed to the effects of the synergist: S.S.S-tributylphosphorotrithioate (DEF) (125 μg/bottle), which inhibits esterase activity. These two synergists were used separately. Approximately 125 mosquitoes were used for each synergist assay. The number of dead or alive mosquitoes was monitored at different time intervals (0, 15, 30, 35, 40, 45, 60, 75, 90,105, 120 minutes). This test allowed us to compare the obtained percentages of dead mosquitoes (Y axis) against time (X axis) before the addition of the synergist to those obtained after the addition of the synergist (Figure 2).

### Statistical analysis and data interpretation

The resistance status of mosquito samples was determined according to the CDC criteria [14,16]. The susceptibility thresholds at the diagnostic time of 30 minutes for both pyrethroids and carbamates are:- Mortality rate = 100%: the population is fully susceptible - Mortality rate < 100%: the population is considered resistant to the tested insecticides. Abbott’s formula was not used in this study for the correction of mortality rates in test-bottles because the mortality rates in all controls was always less than 5% [17]. To appreciate the effects of synergist PBO on *Anopheles gambiae* Agbalilame and Kandi populations resistant to permethrin and deltamethrin respectively and the effect of synergist DEF on *Anopheles gambiae* Tanguieta populations resistant to bendiocarb, we used a Kruskal-Wallis test. The knockdown times for 50% and 95% of tested mosquitoes (kdt50 and kdt95) and mortality times or lethal times for 50% and 95% of tested mosquitoes (LT50 and LT95) were estimated using SPSS version 16.0 (SPSS Inc., Chicago, IL). The significance level was set at 5%.

## Results

### Susceptibility of *Anopheles gambiae s.l.* populations to pyrethroids and to carbamates

The Kisumu strain (control) confirmed its susceptibility status as a reference strain.All female mosquitoes of *Anopheles gambiae* Kisumu that were exposed to CDC bottles treated with permethrin 21.5 μg/bottle, deltamethrin 12.5 μg/bottle and bendiocarb 12.5 μg/bottle were dead and none of them could fly after 30 minutes, which represents the susceptibility threshold time or diagnostic time clearly defined by the CDC protocol. This confirmed that this strain was fully susceptible to these products (Table 1). A large proportion of the *Anopheles gambiae* Agbalilame population (76%) continued to fly again in the bottles following 30 minutes exposure to CDC bottles treated with permethrin. This demonstrated that these populations were highly resistant to this product.

**Table 1:** **Susceptibility status and insecticide resistance levels in *Anopheles gambiae s.l. *populations**

**Populations**	**Insecticides**	**Number tested**	**% Mortality**	**Resistance status**
	Permethrin	37	100	S
Kisumu (Control)	Deltamethrin	110	100	S
	Bendiocarb	111	100	S
Agbalilame	Permethrin	50	24	R
Tanguieta	Bendiocarb	76	78.94	R
Kandi	Deltamethrin	51	43.13	R

Using *Anopheles gambiae* Tanguieta populations, after 30 minutes exposure to CDC bottles treated with bendiocarb, some of these mosquitoes (21.06%) continued to fly again in the bottles.This also demonstrated these populations were resistant to this product. A similar pattern was observed with *Anopheles gambiae* Kandi populations when they were exposed to CDC bottles treated with deltamethrin. Some of these mosquitoes (56.87%) continued to fly again in the bottles.This also demonstrated that these populations were resistant to this product (Table 1).

### Effects of synergist PBO on *Anopheles gambiae* Agbalilame populations resistant to permethrin

**Figure 3:**
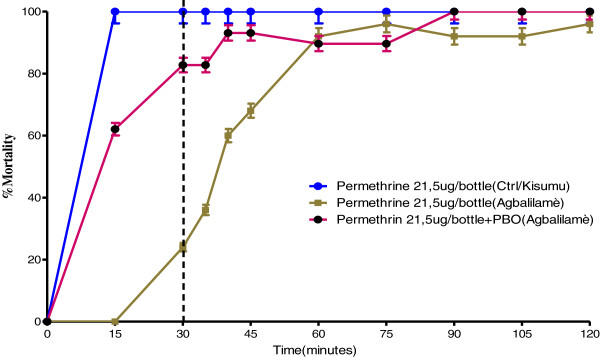
**Implication of mono-oxygenases in resistance of *An. gambiae s.l. *to pyrethroid in Agbalilame locality.**

The analysis of Table 2 and Table 3 shows that after the addition of synergist PBO into CDC bottles treated with permethrin, the KdT50 value obtained with *Anopheles gambiae* Agbalilame populations was 9.32 minutes. This value was lower than the one obtained with permethrin alone which was 43.1 minutes. A similar pattern was observed with the KdT95 value obtained with these same populations, which was 61.54 minutes after the addition of synergist PBO. This value was also lower than the one obtained with permethrin alone, which was 87.6 minutes. Synergist Ratio (SR) (Before addition of PBO/ after addition of PBO) was 4.62 for KdT50 whereas Synergist Ratio (SR) (Before addition of PBO/after addition of PBO) of these same populations was 1.42 for KdT95. The analysis of Figure 3 shows that after the addition of synergist PBO to permethrin 21.5 μg/bottle, the percentage of dead mosquitoes from Agbalilame is higher than the one obtained with permethrin alone. The use of PBO synergist in bottles treated with permethrin 21.5 μg/bottle did not eliminate permethrin resistance, but significantly reduced the level, in point of fact that the mortality rate increased from 24% to 82.75%. These results suggest an implication of mono-oxygenases in resistance of *Anopheles gambiae s.l. *to pyrethroids.

**Table 2:** **Knockdown time KdT50 (minutes) of *Anopheles gambiae *Agbalilame populations to permethrin and permethrin + PBO**

	**Without PBO**		**With PBO**		
**Population**	**Number tested**	**KdT50 (min)**	**Number tested**	**KdT50 (min)**	**SR**
Agbalilame	50	43.1	58	9.32	4.62

**Table 3:** **Knockdown time KdT95(minutes) of *Anopheles gambiae *Agbalilame populations to permethrin and permethrin + PBO**

	**Without PBO**		**With PBO**		
**Population**	**Number tested**	**KdT95 (min)**	**Number tested**	**KdT95 (min)**	**SR**
Agbalilame	50	87.6	58	61.54	1.42

### Effects of synergist DEF on *Anopheles gambiae* Tanguieta populations resistant to bendiocarb

**Figure 4:**
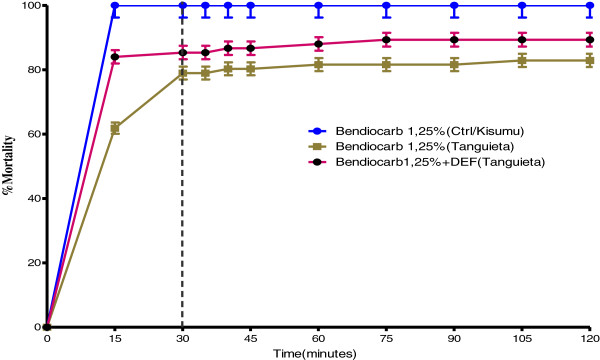
**Implication of esterases in resistance of *An. gambiae s.l. *to carbamates in Tanguieta district.**

The analysis of Tables 4 and 5 shows that after the addition of synergist DEF in CDC bottles treated with bendiocarb, the LT95 value obtained with *Anopheles gambiae* Tanguieta populations was 104.76 minutes. This value was lower than the one obtained with bendiocarb alone which was 137.08 minutes. Synergist Ratio (SR) (Before addition of DEF/after addition of DEF) was 1.30. LT50 value before addition of DEF was 5.23 minutes, whereas LT50 value after addition of DEF with the same *Anopheles gambiae* populations was not determined. The analysis of Figure 4 shows that after the addition of synergist DEF to bendiocarb 12.5 μg/bottle, the percentage of dead mosquitoes from Tanguieta is higher than the one obtained with bendiocarb alone. The use of DEF synergist in CDC bottles treated with bendiocarb 12.5 μg/bottle did not eliminate bendiocarb resistance, but has reduced the level in point of fact that the mortality rate increased from 78.94% to 85.33%. These results suggest an implication of esterases in resistance of *Anopheles gambiae s.l.*to carbamates.

**Table 4:** **Lethal time LT50 (minutes) of *Anopheles gambiae *Tanguieta populations to bendiocarb and bendiocarb + DEF**

	**Without DEF**		**With DEF**		
**Population**	**Number tested**	**LT50 (min)**	**Number tested**	**LT50 (min)**	**SR**
Tanguieta	76	5.23	75	Nd	Nd

Nd: non-determined.

**Table 5:** **Lethal time LT95 (minutes)of *Anopheles gambiae *Tanguieta populations to bendiocarb and bendiocarb + DEF**

	**Without DEF**		**With DEF**		
**Population**	**Number tested**	**LT95 (min)**	**Number tested**	**LT95 (min)**	**SR**
Tanguieta	76	137.08	75	104.76	1.3

### Effects of synergist PBO on *Anopheles gambiae* Kandi populations resistant to deltamethrin

**Figure 5:**
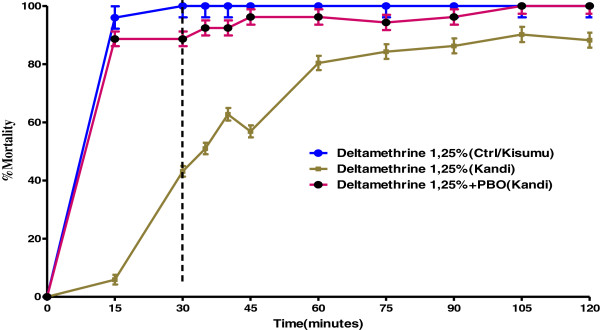
**Implication of mono-oxygenases in resistance of *An. gambiae s.l. *to pyrethroid in Kandi district.**

The analysis of Table 6 and Table 7 shows that after the addition of synergist PBO to CDC bottles treated with deltamethrin, the KdT50 value obtained with *Anopheles gambiae* Kandi populations was 7.79 minutes. This value was lower than the one obtained with deltamethrin alone, which was 43.11 minutes. A similar pattern was observed with KdT95 values obtained with these same populations, which was 51.82 minutes after the addition of synergist PBO. This value was also lower than the one obtained with deltamethrin alone which was 108.37 minutes. Synergist Ratio (SR) (Before addition of PBO/ after addition of PBO) was 5.53 for KdT50, whereas Synergist Ratio (SR) (Before addition of PBO/after addition of PBO) of these same populations was 2.09 for KdT95. The analysis of Figure 5 shows that after the addition of synergist PBO to deltamethrin 12.5 μg/bottle, the percentage of dead mosquitoes from Kandi was higher than the one obtained with deltamethrin alone. The use of PBO synergist in CDC bottles treated with deltamethrin 12.5 μg/bottle did not eliminate deltamethrin resistance, but has significantly reduced the level in point of fact that the mortality rate increased from 43.13% to 88.67%. These results also suggest an implication of mono-oxygenases in the resistance of *Anopheles gambiae s.l. *to pyrethroids.

**Table 6:** **Knockdown time KdT50 (minutes) of *Anopheles gambiae *Kandi populations to deltamethrin and deltamethrin + PBO**

	**Without PBO**		**With PBO**		
**Population**	**Number tested**	**KdT50 (min)**	**Number tested**	**KdT50 (min)**	**SR**
Kandi	51	43.11	53	7.79	5.53

**Table 7:** **Knockdown time KdT95 (minutes) of *Anopheles gambiae *Kandi populations to deltamethrin and deltamethrin + PBO**

	**Without PBO**		**With PBO**		
**Population**	**Number tested**	**KdT95 (min)**	**Number tested**	**KdT95 (min)**	**SR**
Kandi	51	108.37	53	51.82	2.09

## Discussion

Knowledge regarding the level and mechanisms of resistance occurring in a vector population is very important for integrated vector control, in order to decide which control method is effective, efficient, and will not encourage further resistance [18]. Resistance management strategies are mainly based on the rational use of the compounds already available, especially in public health because the number of insecticides is very limited. Resistance to these insecticide families is now widespread in the main malaria vectors *Anopheles gambiae s.l* from Benin. The emergence and spread of *kdr* resistance among *Anopheles gambiae* should burden the large scale programmes of impregnated net distribution that are promoted all over African countries. According to Chandre *et al.*[19], the presence of a*ce-1R* did not necessarily mean that bed-nets impregnated with Carbamates or OP will be ineffective. Similar to Carbamates or OP, the presence of *kdr* mutation in pyrethroid resistance did not necessarily mean that bed-nets impregnated with pyrethroids will be ineffective, because several factors such as the mosquito behavior influence the efficacy of vector control methods. So, control of vector borne diseases uses different methods depending on physiological, behavioural and ecological features of the vector. Insecticide-treated nets (ITNs) also lead to a reduction of human-vector contact by providing a physical barrier.

It is worth mentioning that the locality of Agbalilame is crossed by the Nokoue Lake streams, which sweep and converge several environmental pollutants and pesticide residues from the neighbouring peri-urban cities and farms to the coastal locality of Agbalilame. It is also possible that several ranges of xenobiotics present in these water bodies around Agbalilame might have also contributed to the selection of this resistance in *Anopheles gambiae.* In addition, Padonou and others [20], have found a *kdr* frequency of 0.87 in the M form of *Anopheles gambiae s.s.* from Seme district*.* Further investigations are needed to clearly elucidate the main factors contributing to the high level of resistance recorded in this population of Agbalilame to permethrin. Nevertheless, this resistance to pyrethroids is of great concern for malaria control programs with interventions based on LLINs, as there are high levels of resistance to this insecticide class in Benin. Additionally, there is a risk that if such resistance is not managed properly, it can be further selected by ongoing control interventions such as the pyrethroid impregnated LLINs and IRS to a level that will seriously impact on the success of future control programs. The underlying mechanism of the resistance pattern observed in this population was explored using a synergist assay. The synergist assay with PBO, an inhibitor of Cytochrome P450 monooxygenases, indicated that this enzyme family plays a role in this high permethrin resistance observed in Agbalilame. Indeed, the mortality rate to permethrin was shown to vary significantly when mosquitoes were exposed to PBO. The use of synergist PBO to overcome permethrin resistance in *Anopheles gambiae* Agbalilame showed that this synergist has inhibited mono-oxygenase activity and therefore improved permethrin effectiveness in this *Anopheles gambiae* population.

Kandi is a cotton growing area where various insecticidal products (organophosphates, pyrethroids, etc.) are used to control agricultural pests. Higher activity of mono-oxygenases was also detected in deltamethrin resistance in *Anopheles gambiae* Kandi. This result may reflect an increased selection pressure on malaria vectors due to agricultural practices [21,22]. Based on strong supporting results, several authors [22-25] hypothesized that past and current agricultural use of pyrethroids, DDT and organophosphates for crop protection led to the selection of resistant individuals by challenging larval stages with residual insecticide products accumulating in water bodies around cultivated areas. Indeed, Akogbéto *et al.*[26] demonstrated in Benin that pyrethroid resistant *Anopheles gambiae* larvae reared in water and soil samples taken from vegetable gardens or cotton areas were able to survive and proliferate in contrast to the susceptible phenotype. The underlying mechanism of resistance patterns observed in *Anopheles gambiae* Kandi populations was explored through a synergist assay. The synergist assay with PBO, an inhibitor of Cytochrome P450 monooxygenases, indicated that this enzyme family plays a role in the deltamethrin resistance observed in Kandi. Indeed, the mortality rate to deltamethrin has also significantly varied when mosquitoes are exposed to PBO. The use of synergist PBO to overcome deltamethrin resistance in *Anopheles gambiae* Kandi showed that this synergist has inhibited mono-oxygenase activity and therefore improved deltamethrin effectiveness in this *Anopheles gambiae* population.

Aizoun *et al.*[27] have already reported on the implication of mono-oxygenases in resistance of *Anopheles gambiae* to pyrethroids in Misserete district in southern Benin. Metabolic resistance to pyrethroids is usually associated with increased cytochrome P450 activity [28]. The involvement of P450 enzymes in pyrethroid resistance in the Kenyan population of *Anopheles gambiae* was demonstrated using the P450 inhibitor, piperonyl butoxide (PBO) [9]. Treatment with PBO partially reversed permethrin resistance in this strain although it did not restore susceptibility completely, suggesting that an additional resistance mechanism was involved [29].

*Anopheles gambiae* Tanguieta populations have developed a resistance to bendiocarb after the second round of IRS with bendiocarb (May-June) in Tanguieta district. The bendiocarb resistance recorded in this study raises a special concern for National Malaria Control programs which, because of high resistance to pyrethroids and DDT, are currently introducing bendiocarb based IRS for malaria vector control in West African countries. The use of insecticides for crop protection may explain the level of OP and carbamate resistance observed in some of our rural areas [3]. This is likely the case of bendiocarb resistance observed in *Anopheles gambiae* Tanguieta populations. This resistance is not only due to esterase activity in these *Anopheles gambiae* populations but it is also likely to be due to the *ace-1R* mutation. According to Corbel *et al.*[30], the presence of the *Ace.1* resistance allele at high frequency in neighbouring countries (N’Guessan *et al.*[31]) underlines the need to carefully monitor its extension to Benin. It is difficult to know which mechanisms are operating without conducting a battery of other tests such as genetic analyses. This was beyond the scope of this initial evaluation but further investigations of resistance mechanisms are clearly warranted to better define and quantify resistance mechanisms present in the test populations and verify the preliminary evidence of metabolic-based mechanisms in *Anopheles gambiae* Tanguieta as indicated by bottle bioassays. The underlying mechanism of resistance patterns observed in *Anopheles gambiae* Tanguieta populations was explored through using the synergist assay. The synergist assay with DEF, an inhibitor of esterases, indicated that this enzyme family plays a small role in the bendiocarb resistance observed in *Anopheles gambiae* Tanguieta. Indeed, the mortality rate to bendiocarb has slightly varied when mosquitoes are exposed to DEF. The use of synergist DEF to overcome bendiocarb resistance in *Anopheles gambiae* Tanguieta showed that this synergist has partially inhibited esterase activity and therefore slightly improved bendiocarb effectiveness in this *Anopheles gambiae* population.

Synergists partially restored susceptibility to pyrethroid and carbamate insecticides, and might help mitigate the impact of vector resistance in *Anopheles gambiae* Agbalilame, Kandi and Tanguieta populations. Biochemical assays using synergist results suggested that metabolic resistance is involved in the resistance to pyrethroids and carbamates even if the role of esterases in bendiocarb resistance in *Anopheles gambiae* Tanguieta is small.

## Conclusion

The effects of the synergists PBO and DEF on mosquito populations will be more effective if resistance is only due to biochemical mechanisms that involve the detoxifying enzymes.

The insecticide resistance profile observed in the *Anopheles gambiae* Agbalilame, Kandi and Tanguieta populations highlights the need for further studies to assess the extent and the geographical distribution of this resistance in *Anopheles gambiae* natural populations in Benin and West Africa, as well as a more comprehensive analysis of the resistance mechanisms involved. This will improve the implementation and management of future control programs against this important malaria vector in Benin and in Africa in general.

## Competing interests

The authors declare that there is no conflict of interest.

## Authors’ contributions

MA and NA conceived the study. GGP, RA, RO have participated in the design of the study. Entomologic data was collected by NA, OO, RA and bioassays and laboratory analysis were carried out by NA and OO. FO analyzed the data. MA and NA have participated in the analysis and interpretation of data. VG has contributed to the mapping. The manuscript was drafted by NA and MA has been involved in revision of the manuscript. All authors read and approved the final manuscript.
